# Metabolomic profiling of dried blood spots reveals gender-specific discriminant models for the diagnosis of small cell lung cancer

**DOI:** 10.18632/aging.102670

**Published:** 2020-01-12

**Authors:** Li Yu, Kefeng Li, Xiangmin Li, Chao Guan, Tingting Sun, Xiaoye Zhang

**Affiliations:** 1 Department of Clinical Oncology, Shengjing Hospital of China Medical University, Shenyang, Liaoning 110004, China; 2 School of Medicine, University of California, San Diego, CA 92103, USA

**Keywords:** small cell lung cancer, dried blood spot, metabolomics, differential diagnosis, gender differences

## Abstract

The accurate diagnosis of small cell lung cancer (SCLC) at initial presentation is essential to ensure appropriate treatment. No validated blood biomarkers that could distinguish SCLC from non-small cell lung cancer (NSCLC) has yet been developed. Dried blood spot (DBS) microsampling has gained increasing interest in biomarkers discovery. In this study, we first performed metabolomic profiling of DBS samples from 37 SCLC, 40 NSCLC, and 37 controls. Two gender-specific multianalyte discriminant models were established for males and females, respectively to distinguish SCLC from NSCLC and controls. The receiver operator characteristic (ROC) curve analysis showed the diagnostic accuracy of 95% (95% CI: 83%-100%) in males SCLC using five metabolites in DBS and 94% (95% CI: 74%-100%) for females using another set of five metabolites. The robustness of the models was confirmed by the random permutation tests (P < 0.01 for both). The performance of the discriminant models was further evaluated using a validation cohort with 78 subjects. The developed discriminant models yielded an accuracy of 91% and 81% for males and females, respectively, in the validation cohort. Our results highlighted the potential clinical utility of the metabolomic profiling of DBS as a convenient and effective approach for the diagnosis of SCLC.

## INTRODUCTION

Lung cancer is the most common malignant tumor that threatens human health and one of the leading causes of cancer-related death in both men and women [1]. There are two major subtypes of lung cancer including small cell lung cancer (SCLC) and non-small cell lung cancer (NSCLC). SCLC accounts for about 10–15% of all lung cancer diagnosis. The two subtypes of lung cancer behave differently and thus are treated very differently [2]. Therefore, it is essential for the accurate classification of the subtypes of lung cancer at initial presentation to ensure the selection of appropriate treatment approaches.

Currently, the diagnosis of SCLC primarily relies on the histological and cytological results from tumor tissue biopsies. In many cases, the cytologic features are equivocal due to the sampling issues, fixation artifacts and morphological variability of SCLC tumor cells [3]. Immunohistochemistry (IHC) might be helpful to increase the confidence of the pathologists in SCLC classification. However, a recent study reported that the interobserver agreement for the interpretation of IHC slides was as low as 40% [4]. Moreover, SCLC often originates in the submucosal tissue, which makes the accurate collection of tumor samples challenging [5]. The analysis of tumor markers in biological fluids such as the serum, plasma, and urine has remarkable advantages over histologic differentiation.

The serum level of neuron-specific enolase (NSE) is the tumor biomarker currently used as the reference for the diagnosis, prognosis, and follow-up of SCLC [6]. Unfortunately, recent studies reported that NSE has low sensitivity and the elevated levels of NSE were also observed in about 25% of patients with NSCLC [7]. There is an urgent need for a simple, specific and accurate approach to increase the diagnostic certainty for SCLC.

Metabolites are the small molecules (<2000 Da) with a wide range of functions in cells and organisms such as energy production, signal transduction, and apoptosis. Metabolites are the direct “readout” of the overall physiological status and very sensitive to pathological changes [8]. Metabolomics, the profiling of the metabolites in biofluids, has been increasingly used as a tool for the biomarker discovery and understanding of the pathogenesis of the diseases [9]. However, previous metabolomic studies in lung cancer primarily focused on discriminating lung cancer patients from non-cancer participants [10]. Most of the potential metabolic biomarkers identified in these studies were energy-related metabolites such as glucose and glutamate. These biomarkers were actually reported to be abnormal in other cancers, not limited to lung cancer [10]. There is little information regarding the classification of lung cancer subtypes using circulating metabolite profiles. Recently, with the innovative development of analytical techniques and machine learning-based bioinformatic tools, next-generation metabolomics is now more robust and sensitive allowing the accurate detection of subtle differences between groups [9, 11]. In our previous study, using next-generation metabolomics, we revealed the unique metabolic features in SCLC cells compared to normal human bronchial epithelial cells (HBECs) and NSCLC cells [12].

DBS is a minimally invasive microsampling strategy that involves the collection of a drop of blood on a filter card. Compared to the conventional sampling procedures, DBS has several advantages including the low amount of blood collected, easiness of blood collection, the possibility to store and ship collected samples at room temperature and low cost and logistics [13]. The most common clinical use of DBS is for the screening of inborn errors of metabolism in newborns. In recent years, there is a growing interest in biomarker development and validation based on omic-based analysis of DBS. Metabolomic analysis of DBS has been shown high sensitivity and accuracy for rapid breast [14] and colorectal cancer detection [15]. However, the application of DBS metabolomic approach for the differentiation of lung cancer subtypes has not been reported.

In this study, we conducted a comparative metabolomic analysis of DBS collected from patients with SCLC, NSCLC and healthy controls using liquid chromatography coupled with tandem mass spectrometry (LC-MS/MS). We identified the unique metabolic features in the DBS of both male and female SCLC patients. The selected metabolites biomarkers for distinguishing SCLC from NSCLC and controls were further validated using another cohort.

## RESULTS

### Subjects characteristics

**Figure 1:**
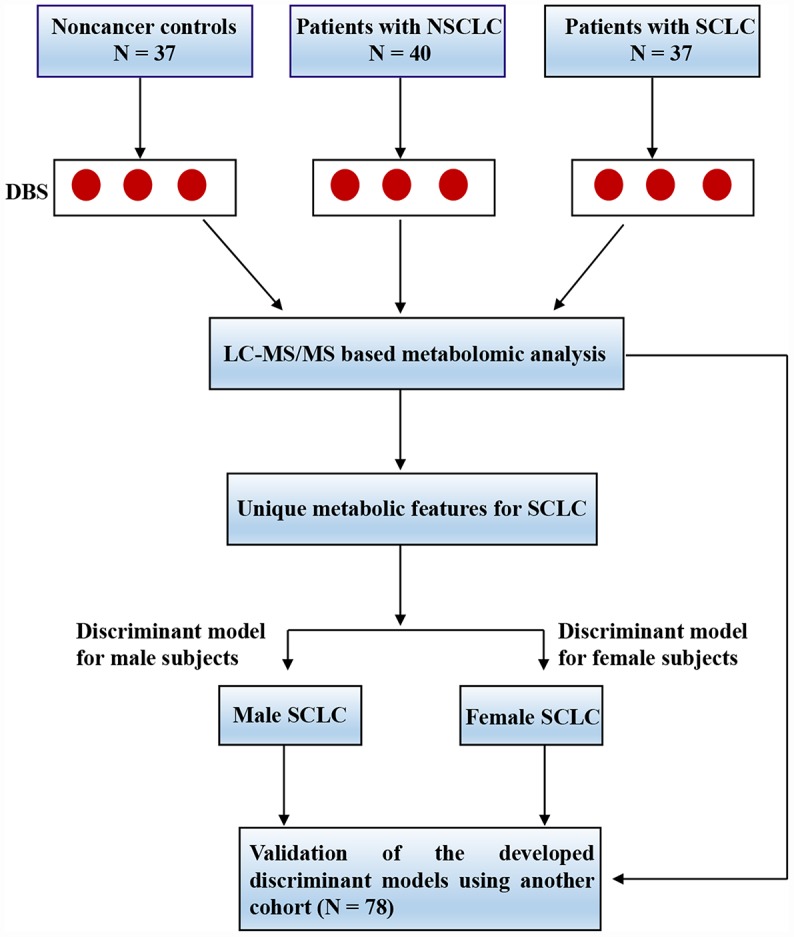
**The experimental flow for the study.** Abbreviations: DBS = dried blood spot; LC-MS/MS = liquid chromatography coupled with tandem mass spectrometry; NSCLC = non-small cell lung cancer; SCLC = small cell lung cancer.

The study flow was shown in Figure 1. In the discovery phase, a total of 114 subjects were recruited including 40 patients with NSCLC, 37 patients with SCLC and 37 noncancer controls (Table 1). In the validation set, 78 cases were enrolled including 20 patients with NSCLC, 31 patients with SCLC and 27 noncancer controls (Supplementary Table 1). The subjects were age and sex-matched and there were no significant differences for gender, smoking status and alcohol drinking record between groups. All the lung cancer patients were at the initial diagnosis prior to any treatment. The details for clinical characteristics were listed in Table 1 and Supplementary Table 1.

**Table 1:** The characteristics of the subjects in the discovery stage.

**Variables**	**Control**	**NSCLC**	**SCLC**	**P value**
N	37	40	37	NA
Age, mean (SD)	62.8 (7.6)	62.6 (9.2)	63.7 (7.6)	0.81
Male (%)	23 (62.6%)	24 (60.0%)	23 (62.6%)	0.73
Smoking Status (%)				
Never	22 (59.5%)	24 (60.0%)	20 (54.1%)	0.95
Former	1 (2.7%)	2(5%)	4 (10.1%)	0.95
Current	14 (37.8%)	14(35%)	18 (48.62%)	0.95
Alcohol drinking status				
Never	29 (78.4%)	32(80.0%)	27 (73.0%)	0.76
Former	0 (0%)	2(5.1%)	4 (17.4%)	0.07
Current	8 (21.6%)	6(15.0%)	6 (16.2%)	0.75

Abbreviations: SCLC = small cell lung cancer; SD = standard deviation; NSCLC = non-small cell lung cancer.

### The metabolomic profiling of SCLC in the DBS of the discovery set

To investigate the unique metabolic features of SCLC, we first performed comparative metabolomic profiling of DBS for the discovery cohort. After quality control analysis, we detected and identified 372 metabolites with less than 20% miss values in all the samples. The representative chromatogram from one of the DBS specimens is shown in Supplementary Figure 1. Multivariate analysis showed the metabolomic profile of SCLC in DBS was not separated from NSCLC and noncancer controls by PLS-DA when all the male and female samples were combined for analysis (Supplementary Figure 2A). In addition, ROC curve analysis revealed low diagnostic accuracy for SCLC if the males and females were analyzed together (Supplementary Figure 2B). Previous studies reported that women have a different incidence of SCLC compared to men [16, 17]. These results suggested that there might be gender differences in metabolomic profiles between male and female SCLC patients. We then analyzed the male and female samples separately.

**Figure 2:**
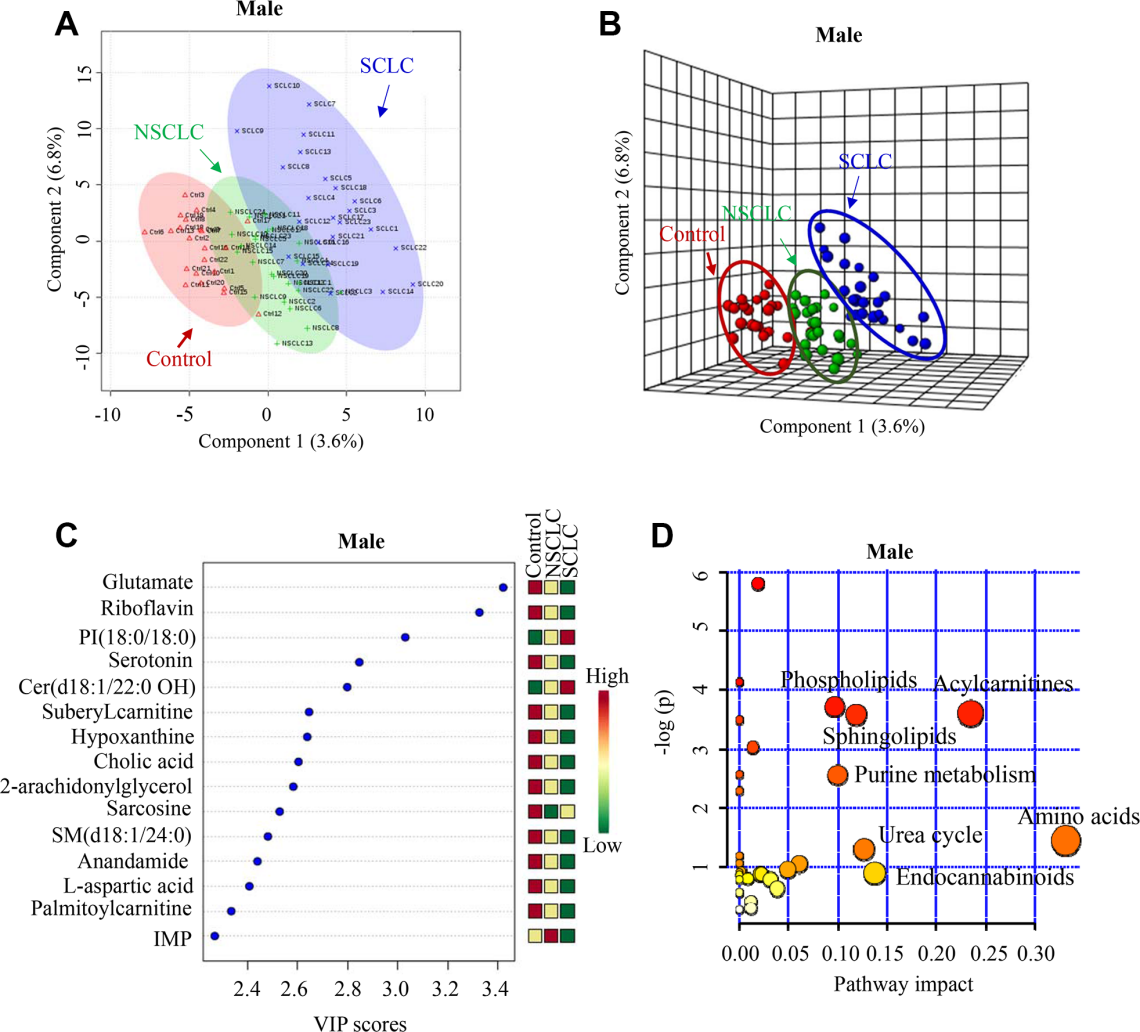
**The unique metabolic features of male SCLC compared to NSCLC and controls.** (**A**, **B**) Multivariate analysis of metabolomic data using PLS-DA resulted in a clear separation of metabolic features among SCLC, NSCLC and the control group in males. (**A**) 2-D plot. (**B**) 3-D plot. (**C**) The top 15 most differential metabolites in male patients with SCLC revealed by VIP analysis. VIP>1.5 was considered as statistically significant. The VIP results were also verified by univariate ANOVA analysis. (**D**) The top pathways disturbed in male SCLC patients. Abbreviations: IMP = inosine monophosphate; Cer = Ceramide; SM = Sphingomyelin; PI = Phosphatidylinositol; NSCLC = non-small cell lung cancer; PLS-DA = partial least square discriminant analysis; SCLC = small cell lung cancer; VIP = variance in projection.

Both 2-D and 3-D PLS-DA plots showed that male SCLC had a unique metabolic signature that was distinct from patients with NSCLC and noncancer controls (Figure 2A and 2B). Since PLS-DA is a supervised model, we then performed leave-one-out cross-validation (LOOCV) to evaluate the level of overfitting for the PLS-DA model (Supplementary Figure 3A). The Q^2^ value of 0.76 indicated the PLS-DA model is a good predictive model for the dataset. The top 15 most discriminant metabolites revealed by variance in projection (VIP) analysis after PLS-DA was listed in Figure 2C. The VIP metabolites were further confirmed by univariate one-way ANOVA analysis. Pathway analysis revealed that sphingolipids metabolism, phospholipids, purine, urea cycle, acylcarnitines, amino acids, and endocannabinoids metabolism were significantly altered in patients with SCLC compared to NSCLC and controls (Figure 2D).

**Figure 3:**
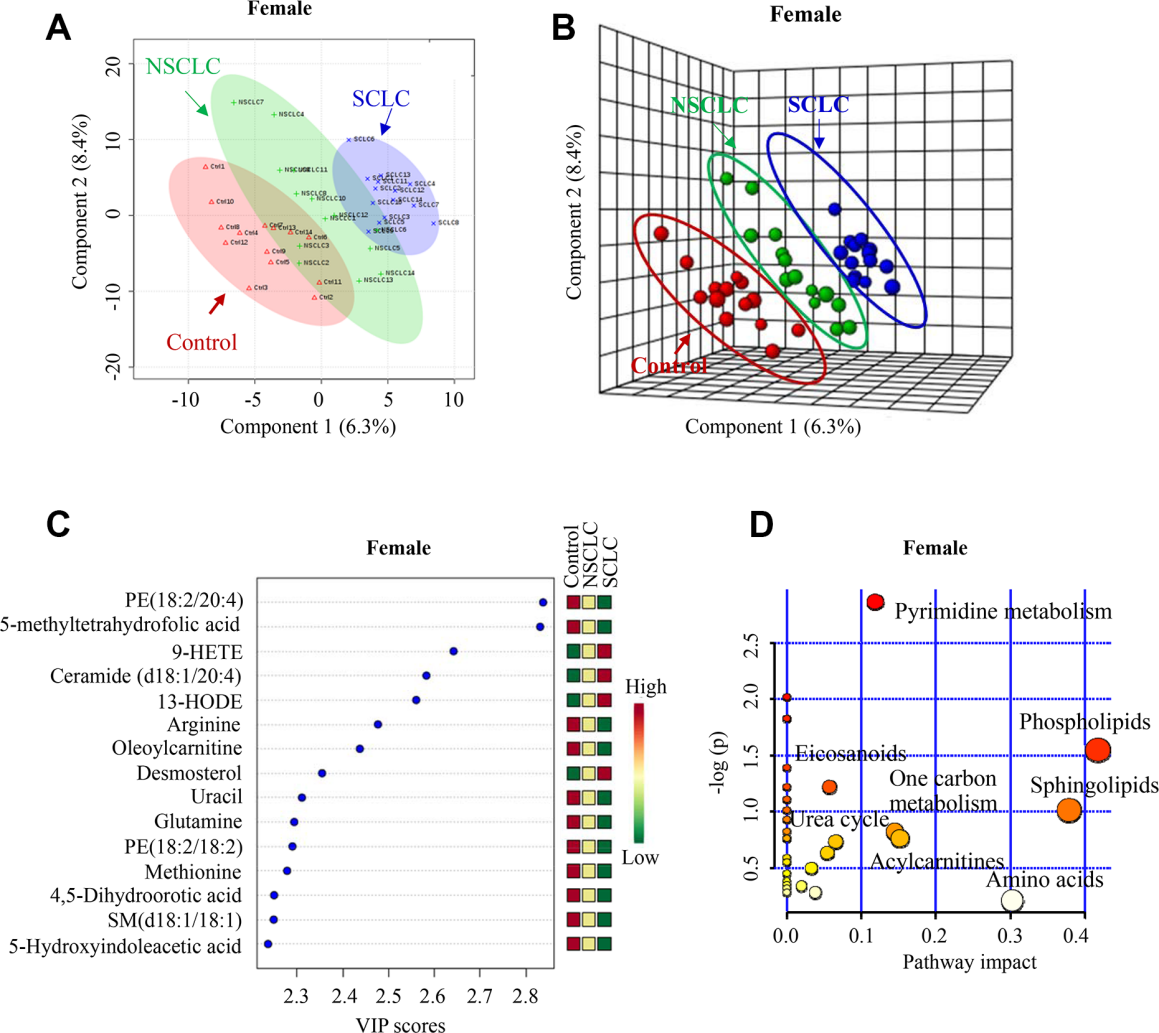
**The unique metabolic features in the DBS of female SCLC patients compared to NSCLC and noncancer controls.** (**A**, **B**) Multivariate analysis of metabolomic data using PLS-DA resulted in a clear separation of metabolic features among SCLC, NSCLC and the control group in females. (**A**) 2-D plot. (**B**) 3-D plot. (**C**) The top 15 most differential metabolites in female patients with SCLC revealed by VIP analysis. VIP>1.5 was considered as statistically significant. The VIP results were also verified by univariate ANOVA analysis. (**D**) The top pathways disturbed in female SCLC patients. Abbreviations: DBS = dried blood spot; NSCLC = non-small cell lung cancer; PE = phosphatidylethanolamine; 9-HETE = 9-hydroxyeicosatetraenoic acid; 13-HODE = 13-Hydroxyoctadecadienoic acid; SM = Sphingomyelin; PLS-DA = partial least square discriminant analysis; SCLC = small cell lung cancer; VIP = variance in projection.

Similarly, there was a clear separation of metabolomic features in DBS among female SCLC, NSCLC and control subjects revealed by both 2-D and 3-D plots (Figure 3A and 3B). The PLS-DA classification model for the female dataset was confirmed by LOOCV (Supplementary Figure 3B). The top 7 most differential metabolites in the VIP analysis were from the biochemical pathways of phospholipids (Phosphatidylethanolamine, PE (18:2/20:4)), one-carbon metabolism (5-methyltetrahydrofolic acid), eicosanoids (9-HETE), sphingolipids (Ceramide (d18:1/20:4)), acylcarnitines (Oleoylcarnitine), branch chain amino acids (Arginine) and urea cycle (Glutamate) (Figure 3C and 3D).

### The unique metabolic signatures in the DBS of male and female SCLC

**Figure 4:**
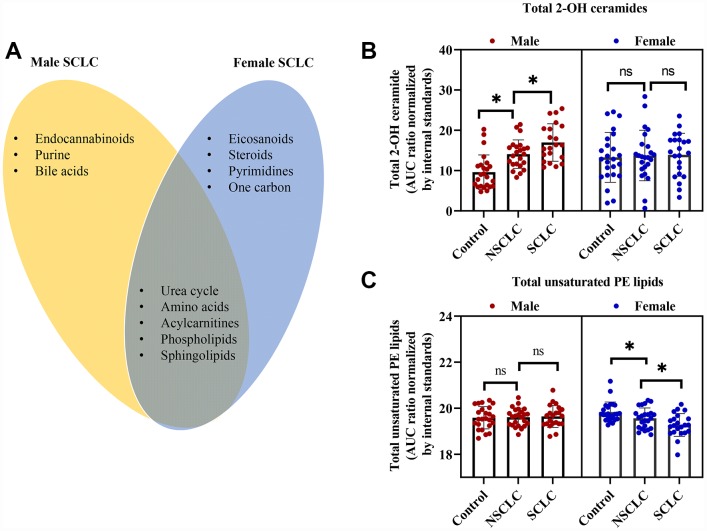
**The shared and unique metabolic signature between male and female patients with SCLC.** (**A**) The common and unique disturbed pathways in male and female SCLC compared to NSCLC and controls. (**B**) Total 2-hydroxy (2-OH) ceramides were significantly higher in male SCLC, while, no differences were observed in female patients with SCLC. (**C**) Total unsaturated PE was dramatically lower in female SCLC without significant changes in male SCLC. One-way ANOVA followed by Tukey’s test. * P < 0.05 and ns: nonsignificant. Abbreviations: NSCLC = non-small cell lung cancer; PE = phosphatidylethanolamine; SCLC = small cell lung cancer.

We then compared the shared and unique metabolic features in male and female SCLC. As shown in the Venn diagram (Figure 4A), endocannabinoids, purine and bile acids metabolism were only significantly altered in male SCLC, while, eicosanoids, steroids, pyrimidines, and one-carbon metabolism were disturbed in female SCLC patients. Five disturbed metabolic pathways including sphingolipids, phospholipids, acylcarnitines, amino acids, and urea cycle were common to both males and females with SCLC (Figure 4A).

Even though sphingolipids and phospholipids were both altered in male and female SCLC patients, further analysis showed gender-specific differences in the subclasses of these two classes of lipids. In detail, the levels of total 2-hydroxy ceramides in DBS of male SCLC were significantly higher than male NSCLC and controls (Figure 4B). But there were no significant differences in 2-hydroxy ceramides between female subjects. The changes in the DBS of female SCLC were mainly unsaturated ceramides such as Ceramide (d18:1/20:4) (Figure 3C). Additionally, in the subclasses of phospholipids, the unsaturated PE lipids decreased dramatically only in the DBS of female SCLC (Figure 4C), while, the saturated phosphatidylinositol lipids increased significantly in male SCLC (Figure 2C).

### The discriminant models of SCLC for male and female patients based on the discovery set

We next aimed to build the classification models that could be used for the identification of SCLC. We first evaluated the feasibility of the univariate model using male samples. Surprisingly, we found that a single analyte (2-arachidonylglycerol, 2-AG) had an accuracy of 85.6% (95% CI: 72.4%-95.1%) for the diagnosis of SCLC in the discovery cohort (Supplementary Figure 4A). However, it performed poorly in the validation cohort (Supplementary Figure 4B). This suggested that single metabolic biomarker might be biologically implausible for complex diseases like cancer.

We then explored the utility of the multiple variables classification. A set of 3-11 metabolites were selected and tested according to the following criteria: (1) Top 30 VIP metabolites in PLS-DA model (2) Covering as many as different biochemical pathways and k-mean clusters (3) High frequency in the least absolute shrinkage and selection operator (LASSO) model. By using this approach, the biological variation across the subjects is more readily accommodated. The performance of each classifier set of metabolites was evaluated by ROC analysis. The optimal variable combinations were obtained using the forward selection and backward elimination methods [18].

**Figure 5:**
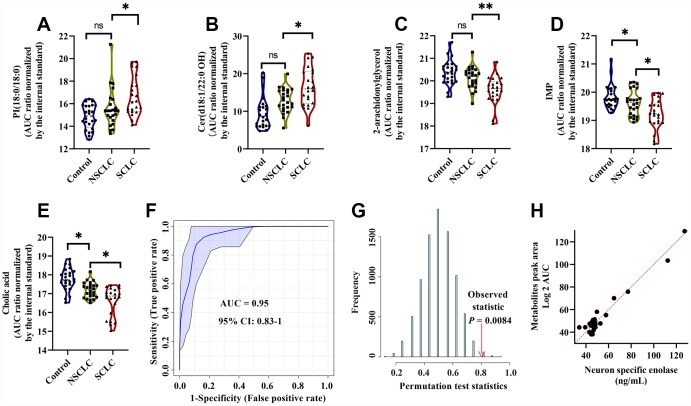
**The diagnostic performance of the developed multianalyte discriminant model for male SCLC in the discovery set.** (**A**–**E**) Violin plots showed the relative levels of five selected metabolites in the model. (**A**) PI(18:0/18:0); (**B**) Cer(d18:1/22:0 OH); (**C**) 2-Arachidonoylglycerol; (**D**) IMP; and (**E**) Cholic acid. The AUC ratio for each metabolite was calculated by the AUC of the corresponding internal standard. One-way ANOVA followed by Tukey’s test was used. *P < 0.05 and ns: nonsignificant. (**F**) ROC curve analysis for distinguishing male SCLC patients from NSCLC and controls. (**G**) The random permutation test to examine the robustness of the model. Permutation P-value represented the probability that the classification of SCLC and NSCLC and controls using the selected metabolites could be obtained by chance. (**H**) Multiple linear regression analysis showed a significant correlation between the five selected metabolites and serum neuron-specific enolase (NSE) in male SCLC patients. R^2^ = 0.63, P < 0.01. Abbreviations: IMP = inosine monophosphate; PI = Phosphatidylinositol; Cer = Ceramide; NSCLC = non-small cell lung cancer; NSE = neuron-specific enolase; ROC = receiver operator characteristic; SCLC = small cell lung cancer.

As a result, a 5-metabolite panel including 2-AG, cholic acid, PI (18:0/18:0), IMP and Cer (d18:1/22:0 OH) was selected as the best variable model for male SCLC. The biochemical pathways, VIP scores in the PLS-DA model, k-mean clusters and frequency of LASSO model of the selected metabolites were listed in Supplementary Table 2. The relative levels of PI (18:0/18:0) and Cer (d18:1/22:0 OH) were significantly upregulated in male SCLC (Figure 5A, 5B). In contrast, 2-arachidonylglycerol, IMP and cholic acid decreased significantly in the DBS of male SCLC subjects (Figure 5C–5E). The ROC analysis using the 5-metabolite panel yielded the diagnostic accuracy of 0.95 (95% CI: 0.83-1) for the diagnosis of male SCLC patients in the discovery set (Figure 5F). The selected 5-metabolite classifier was further validated by a random permutation test (500 times). A P value of 0.0084 indicated the classifier was robust (Figure 5G). The relative importance of each metabolite in the classification model of SCLC calculated by multinomial logistic regression analysis is listed in Supplementary Table 4. Among 5 selected metabolites in the model, Cholic acid has the highest odds ratio of 2.48 (95% CI: 2.19 - 2.76) for SCLC over the controls and NSCLC. In addition, multiple linear regression analysis showed a significant correlation between the serum levels of NSE and the relative levels of 5 diagnostic metabolites in the DBS of male SCLC subjects (R^2^ = 0.63, P < 0.01, Figure 5H).

**Figure 6:**
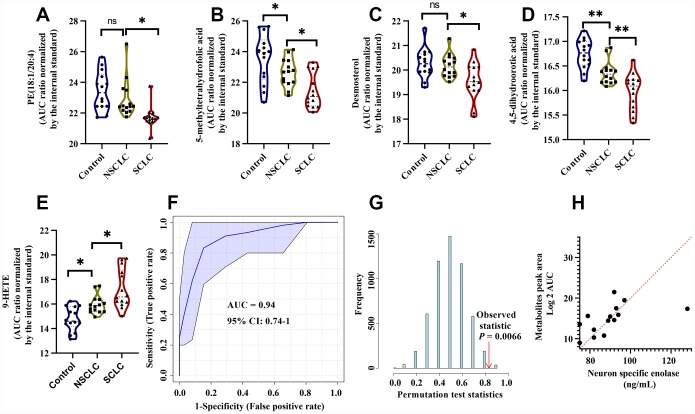
**The diagnostic performance of the developed multianalyte discriminant model for female SCLC in the discovery set.** (**A**–**E**) Violin plots showed the relative levels of five selected metabolites in the female discriminant model. (**A**) PE (18:1/20:4); (**B**) 5-Methyltetrahydrofolic acid; (**C**) Desmosterol; (**D**) 4, 5-Dihydroorotic acid and (**E**) 9-HETE. (**F**) ROC curve analysis for distinguishing female SCLC patients from female NSCLC and controls. (**G**) The random permutation test to examine the robustness of the female discriminant model. (**H**) Multiple linear regression analysis showed a significant correlation between the five selected metabolites and NSE in female SCLC patients. R^2^ = 0.56, P < 0.05. Abbreviations: NSCLC = non-small cell lung cancer; PE = phosphatidylethanolamine; 9-HETE = 9-hydroxyeicosatetraenoic acid; ROC = receiver operator characteristic; SCLC = small cell lung cancer.

The discriminant model for female SCLC was established on the basis of another set of metabolites including PE (18:1/20:4), 5-Methyltetrahydrofolic acid, Desmosterol, 4, 5-Dihydroorotic acid, and 9-HETE. There biochemical pathways, VIP scores in the PLS-DA model, k-mean clusters and frequencies of the LASSO model were shown in Supplementary Table 3. The levels of PE (18:1/20:4), 5-methyltetrahydrofolic acid, Desmosterol, and 4, 5-Dihydroorotic acid were dramatically reduced in female SCLC compared to the controls and NSCLC (Figure 6A–6D). In contrast, 9-HETE was significantly higher in female SCLC (Figure 6E). In the discovery set, the ROC analysis using the selected analytes in DBS had the diagnostic accuracy of 0.94 (95% CI: 0.74-1) for the diagnosis of female SCLC patients (Figure 6F). Classifier robustness for the model was verified with a random permutation test with a P value of 0.0066 (Figure 6G). We then performed the multinomial logistic regression analysis to elucidate the relative importance of each metabolite for the classification of SCLC in females (Supplementary Table 5). We found that among five selected metabolites in the female model, the level of Desmosterol in DBS of SCLC has the highest an odds ratio (3.35, 95% CI: 3.06 - 3.63) over the controls and NSCLC. In addition, similar to the male classifier, a significant correlation with NSE levels in the serum was found by multiple linear regression analysis (R^2^ = 0.56, P < 0.05, Figure 6H).

### Validation of the discriminant models

To validate the accuracy of the developed models, we conducted the metabolomic profiling of another 78 DBS specimens (Supplementary Table 1). For the male subjects, the discriminant model established from the discovery set distinguished male SCLC group from those of in the validation set with the sensitivity of 86.6% (95% CI: 59.5%-98.3%) and the specificity of 84.1% (95% CI: 63.9%-95.5%) (Supplementary Table 6). Similarly, for the female subjects, the selected 5 metabolites from the discovery cohort had the sensitivity of 86.4% (95% CI: 65.1%-97.1%) and the specificity of 84.2% (95% CI: 68.8%-97.1%) for distinguishing female SCLC from the controls and NSCLC in the validation set (Supplementary Table 6).

## DISCUSSION

Even though a few protein biomarkers in the serum have been used for lung cancer diagnosis, biomarkers capable of distinguishing lung cancer subtypes are yet to be discovered [19]. In this study, we demonstrated the unique metabolomic characteristics in DBS of SCLC patients compared to NSCLC and controls. We developed two highly sensitive and specific discriminant models for the diagnosis of male and female SCLC, respectively. The models were further verified with an independent validation cohort using the same analytic method as that in the discovery set. The selected five-metabolite panels of biomarkers from DBS offer a simple and minimally invasive approach that may facilitate the accurate diagnosis and potentially lead to the effective and appropriate treatment of SCLC.

We identified the clear separation of DBS metabolomic profiles by PLS-DA between SCLC, NSCLC and the controls in both male and female subjects. This phenomenon was similar to our previous findings in the metabolomic profiling of SCLC tumor cells [12]. Out of the top 30 discriminating metabolites detected in DBS, only 6 were significantly altered in SCLC tumor cells. This might be due to the differences in the compositions between the whole blood and cells. In addition to red, white cells and platelets, the whole blood contains extracellular metabolites from the plasma. In contrast, only intracellular metabolites were measured in SCLC tumor cell lines. However, we found that even though the discriminating metabolites were largely different, the differential metabolic pathways in SCLC were highly similar in DBS and tumor cell lines. This highlighted the presence of “metabostasis” or metabolic balance that exists between metabolites in cells, plasma, and tissues and DBS could potentially reflect the abnormalities in lung cancer tissues [20].

The present study reported for the first time the gender-specific biomarkers for distinguishing SCLC from controls and NSCLC. This suggested that gender differences might need to be considered in the diagnosis and treatment of SCLC patients. Similarly, metabolic biomarker profiles were reported to be significantly different between men and women in cardiovascular diseases (CVD) [21] and chronic obstructive pulmonary disease (COPD) [22]. A previous study had also reported some sex-related differences in plasma proteins in NSCLC patients [23]. For example, the plasma level of soluble Fas (sFas) was significantly elevated in men with NSCLC but not in women. The reasons for the presence of the gender differences in SCLC remain largely unknown and might be associated with the differences in sex hormones and the proportion of fat tissue. In this study, we found a dramatic decrease in PE lipids in female SCLC. It was reported that estrogen induces the upregulation of phosphatidylethanolamine N-methyltransferase (PEMT), a key enzyme in the conversion of PE lipids to phosphatidylcholine [24]. Another study showed that estradiol was closely related to the enzymes and proteins linked to enzyme metabolism pathways and lipids biosynthesis [25]. Additionally, testosterone production was suppressed by 2-hydroxy ceramides [26]. The role of sex hormones, however, does not fully explain the metabolic differences between men and women. There are fundamental differences in the regulation of metabolic homeostasis between the sexes [27]. Further studies are needed on the metabolic differences between men and women with SCLC.

Through the selection and validation in two cohorts, we identified five metabolites for SCLC diagnosis in males including PI (18:0/18:0), Cer (d18:1/22:0 OH), 2-AG, IMP and Cholic acid. We found a significant increase of PI lipids and 2-hydroxy ceramides in the DBS of male SCLC patients compared to NSCLC and the noncancer controls. PI lipids are not only key cell membrane constituents but also involved in essential metabolic processes such as cell proliferation, growth, and survival via the activation of PI3K/AKT/mTOR signaling pathway [28]. Interestingly, an increase of PI lipids was also observed in the tumor tissue of NSCLC and lymphomas compared to normal tissue [29, 30]. The roles of ceramides and 2-hydroxy ceramides in cancer are not fully understood. Studies in HeLa cells revealed that overaccumulation of C24 ceramide protected cells from ionizing radiation (IR)-induced apoptosis [31]. A strong negative correlation between bile acids and membrane receptor TGR5 was recently reported and the overexpression of TGR5 was found to be associated with cell growth and migration in NSCLC [32]. Endocannabinoids including 2-AG and anandamide were known to be involved in cancer tumor growth and progression and associated pain [33]. However, the direction of the changes for endocannabinoids in different tumors was not consistent. Sailler and the co-authors found the elevated level of circulating 2-AG in the plasma of patients with brain tumor and breast cancer [34]. In our study, we found a decrease of 2-AG in the DBS of SCLC patients. This might be due to the differential roles of endocannabinoids in different cancers and different cell types [35].

In female SCLC patients, the circulating metabolites in DBS including PE (18:1/20:4), 5-methyltetrahydrofolic acid, Desmosterol, 4, 5-dihydroorotic acid, and 9-HETE were significantly altered. 5-methyltetrahydrofolic acid plays an important role in DNA synthesis, repair, methylation, and chromosomal integrity. Previous studies showed that the polymorphisms of 5-methylenetetrahydrofolate reductase (MTHFR) gene were associated with the risk of lung cancer in the female population [36, 37]. Beyond the literature, in this study, we found a significant reduction of 5-methyltetrahydrofolic acid in female SCLC patients. 9-HETE is an eicosanoid metabolite of arachidonic acid and the elevated level of 9-HETE was observed in DBS of women SCLC patients in our study. The link between 9-HETE and SCLC was unknown. However, the activation of 20-HETE biosynthesis was reported to promote the proliferation of cancer cells [38].

We have not tested the prognostic value of two developed discriminant models for small cell lung cancer. Some of the selected biomarker metabolites have been reported to be associated with the prognosis of several other diseases in the previous studies. For example, plasma ceramides are the potential prognostic biomarkers for acute myocardial infarction (MI) and sepsis [39, 40]. Plasma 5-Methyltetrahydrofolic acid is associated with the overall survival of postmenopausal breast cancer patients [41]. The circulating PE lipids were significantly correlated with poor prognostic genotypes of human papillomavirus in cervical cancer [42]. To our knowledge, the prognostic role of the multi-biomarker models in the whole has not been tested in the literature.

## CONCLUSIONS

In summary, we demonstrated the presence of unique metabolic biosignature in the DBS of SCLC patients compared to NSCLC and controls. The gender-specific discriminant models with high accuracy for SCLC were developed and validated. Our study highlighted the potential clinical utility of the metabolomic profiling of DBS as a convenient and feasible approach to help the better diagnosis of SCLC. Further validation in larger cohorts will be needed through multi-regional and multi-center studies.

## MATERIALS AND METHODS

### Subjects and sample collection

This study was registered at the China Clinical Trial Registration Center (ID: ChiCTR-DDD-17010893) (http://www.chictr.org.cn/showproj.aspx?proj=18317). The study protocol was approved by the Ethics Committee of Shengjing Hospital of China Medical University on February 22, 2017. The study was conformed to the rules of the Declaration of Helsinki of 1957: Ethical Principles for Medical Research Involving Human Subjects. Written informed consent was obtained from all the subjects after full disclosure of the study details.

Inclusion criteria: volunteers in the control group met the following criteria: (1) no significant diseases (cancer, diabetes, cardiovascular disease, etc.), or recovery for more than 3 months; (2) no long-term medication history; (3) no surgery in the last 3 months; (4) age and gender-matched with SCLC patients. Patients in the SCLC and NSCLC group met the following criteria: (1) at the initial diagnosis prior to the treatment; (2) a pathological diagnosis of SCLC or NSCLC. In the discovery stage, a total of 114 subjects were enrolled and in the validation stage, another 78 subjects were recruited. The subjects’ characteristics were listed in Table 1 and Supplementary Table 1.

Blood collection and DBS preparation: the finger-prick blood was directly spotted onto Whatman 903 Specimen Collection Paper Cards (GE Healthcare, USA). Three spots were taken from each subject and the spots were dried completely and stored at room temperature with desiccant before analysis. All the specimens were fasting blood samples.

### Extraction of metabolites from DBS

The extraction of metabolites from DBS was conducted according to a previous study with modifications [43]. Briefly, a 3 mm disc per subject was obtained using a GE Healthcare Uni-Core punch and transferred to a 1.5 mL tube. A blank disc from the adjacent of DBS from the same card was used as the analytical blank. One hundred ninety (195) μL of extraction buffer (isopropanol/acetonitrile/water, 3:3:2, v/v/v) and 5 μL of internal standard mix (custom-synthesized stable isotope cocktail) [44] were added to each tube. The mixture was vortexed thoroughly and incubated on ice for 10 min. Samples were centrifuged at 16,000 g for 10 min at 4 °C. The supernatant was stored at -80 °C prior to metabolomic analysis.

### LC-MS/MS-based metabolomic analysis

The metabolomic analysis was performed on a Shimadzu LC-20AD (Shimadzu, Japan) coupled with a Qtrap 5500 mass spectrometer (SCIEX, USA) as described before with slight modifications [44, 45]. Briefly, the chromatographic separation was achieved on a Luna NH2 column (250 × 2 mm, 5 μm, Phenomenex, USA) with the following conditions: mobile phase A: 95% H_2_O + 5% acetonitrile + 20 mM ammonium hydroxide + 20 mM ammonium acetate (pH 9.4); B: 100% acetonitrile; the flow rate: 0.35 mL/min, the column temperature: 25 °C. The injection volume was 10 μL. The gradient was: 0-3 min 95% B, 3-6 min 75% B, 6-7 min 0% B, 7-15 min 0% B, and 15-17 min, 95% B. MS/MS analysis was performed by scheduled multiple reaction monitoring (sMRM) with electrospray ionization (ESI) and positive and negative switch mode. The polarity switch time was set at 50 ms. The ion source temperature was 500 °C. A total of 420 metabolites covering the major metabolomic pathways were targeted.

The chromatographic peaks were integrated using MultiQuant 3.0 (SCIEX, USA) and confirmed by manual inspection. All the detected metabolites were normalized using the corresponding internal standards and the AUC ratios (AUC of the detected metabolites/AUC of the internal standards) prior to the statistical analysis.

### Statistical analysis

The general statistical analysis was performed using GraphPad Prism 8.0 (GraphPad, USA). Data were mean and standard deviation (SD) for continuous variables and actual values with percentages for categorical variables. One-way ANOVA followed by Tukey’s test was used for multiple comparisons of continuous variables and two proportion z test was used for the categorical variables. Multiple linear regression analysis was performed to analyze the relationship between serum NSE and the selected metabolites biomarkers. Significance was set at P < 0.05.

Metabolomic analysis was performed using metaboanalyst 4.0 (https://www.metaboanalyst.ca). The k-nearest neighbor method was used to deal with missing data due to individual differences, and the metabolites with missing data > 50% were removed. The data was log 2 transformed and auto-scaled. Multivariate analysis was conducted using PLS-DA. Since PLS-DA is a supervised model, the accuracy of the PLS-DA model was verified by LOOCV and Q^2^ values were reported which represented the estimate of the predictive ability for the models. The top 30 most influential metabolites were obtained by VIP. VIP scores > 1.5 were considered statistically significant. The metabolites were also verified by a one-way ANOVA test. The significant metabolites identified by VIP analysis was then used for biochemical pathway analysis. In addition to the PLS-DA model, both the unsupervised k-mean clustering model and the LASSO frequencies were also performed.

Sets of 1-11 metabolites were manually selected by bootstrap resampling using the VIP lists and pathways obtained PLS-DA model, k-mean clusters and LASSO frequencies as an example of one possible multianalyte diagnostic classifier. The diagnostic performance of the selected classifiers was evaluated using ROC implemented in Metaboanalyst 4.0. In order to produce a smooth ROC curve, 100 cross-validations (CVs) were performed and the results were averaged to generate the plots. Classifier robustness was estimated by the permutation test (500 times). The sensitivity, specificity, accuracy, positive predictive value (PPV) and negative predictive value (NPV) of the selected biomarkers were analyzed by 2 × 2 contingency table analysis. The relative importance of each metabolite in the predictive models was evaluated using multinomial logistic regression analysis. The multinomial logistic regression coefficients, P values, and odds ratios were reported.

## Supplementary Material

Supplementary Figures

Supplementary Tables
