# Supplementary material for: Metabolomic profiling of dried blood spots reveals gender-specific discriminant models for the diagnosis of small cell lung cancer
Source: Aging (Albany NY). 2020 Jan 12;12(1):978–95. doi: 10.18632/aging.102670 (PMC6977651; doi:10.18632/aging.102670)
Supplement: Supplementary Figures [file aging-12-102670-s002..pdf]

SUPPLEMENTARY FIGURES

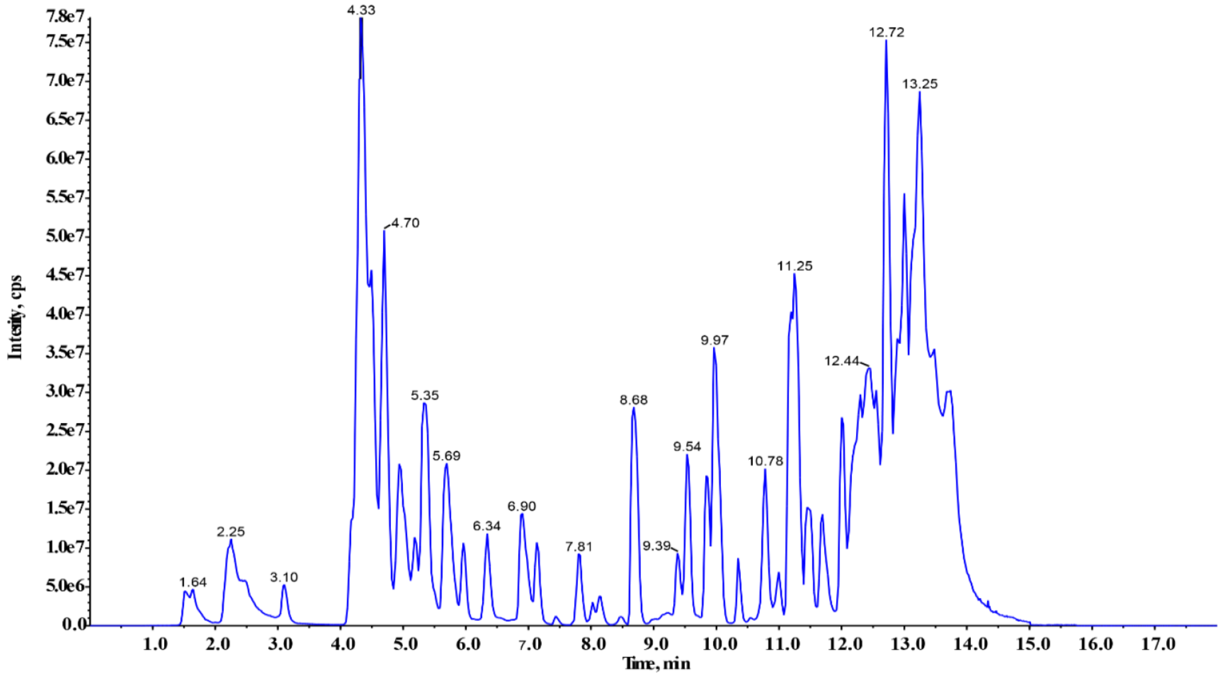

Supplementary Figure 1. The representative chromatogram of the metabolomic profiling of a dried blood spot (DBS) specimen.

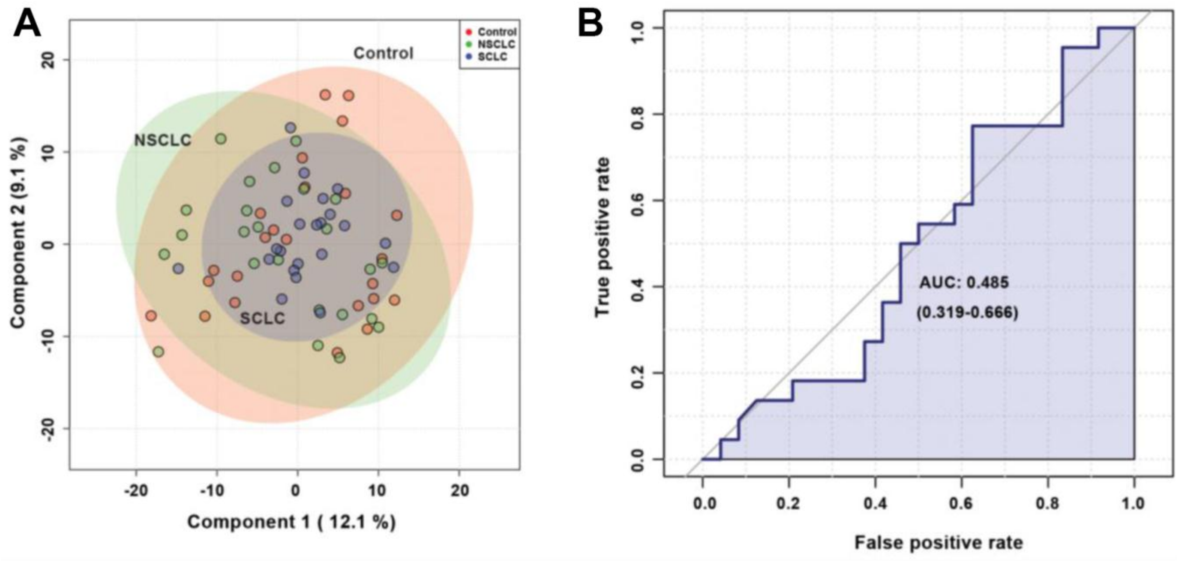

Supplementary Figure 2. No unique metabolic features were discovered to distinguish SCLC from controls and NSCLC when male and female samples were combined for data analysis. (**A**) The metabolomic profile of SCLC was not separated from the controls and NSCLC by PLS-DA when all the samples were analyzed together. (**B**) ROC curve analysis showed low diagnostic accuracy for SCLC when males and females were combined. **Abbreviations**: NSCLC = non-small cell lung cancer; PLS-DA = partial least square discriminant analysis; ROC = receiver operator characteristic; SCLC = small cell lung cancer.

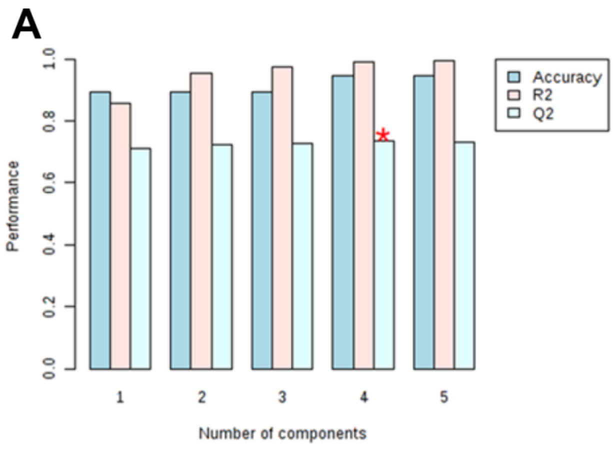

Supplementary Figure 3. LOOCV confirmed that the PLS-DA models were able to effectively and accurately discriminate SCLC patients from NSCLC and controls in male and female subjects, respectively. (**A**) male subjects; (**B**) female subjects. Abbreviations: LOOCV = leave-one-out cross-validation; NSCLC = non-small cell lung cancer; PLS-DA = partial least square discriminant analysis; SCLC = small cell lung cancer.

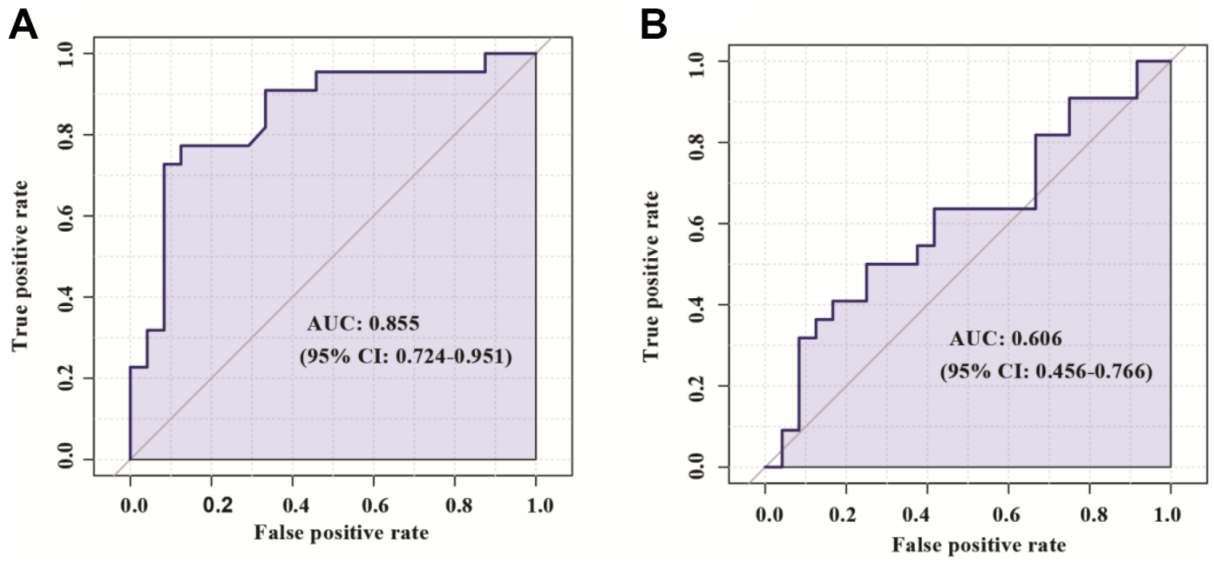

Supplementary Figure 4. The classification performance of the univariate model for the diagnosis of SCLC in male subjects. (**A**) The discovery set; (**B**) Validation set. The single analyte used in the figure was 2-arachidonylglycerol. Abbreviations: SCLC = small cell lung cancer.
