# Supplementary material for: Metabolomic profiling of dried blood spots reveals gender-specific discriminant models for the diagnosis of small cell lung cancer
Source: Aging (Albany NY). 2020 Jan 12;12(1):978–95. doi: 10.18632/aging.102670 (PMC6977651; doi:10.18632/aging.102670)
Supplement: Supplementary Tables [file aging-12-102670-s001..pdf]

SUPPLEMENTARY TABLES

Supplementary Table 1. The characteristics of the subjects in the validation cohort.

Variables	Control	NSCLC	SCLC	P value
N	27	20	31	NA
Age	61.5 ± 5.4	62.1 ± 4.3	62.9 ± 6.1	0.62
Male (%)	13 (48.2%)	12 (60%)	15 (48.4%)	0.61
Smoking Status (%)				
Never	13 (48.1%)	9 (45.0%)	14 (45.2%)	0.99
Former	2 (7.4%)	3 (15.1%)	4 (10.1%)	0.72
Current	12 (44.4%)	8 (40.0%)	13 (41.9%)	0.99
Alcohol drinking status				
Never	15 (55.6%)	12 (60.0%)	16 (51.6%)	0.99
Former	3 (11.1%)	2 (10.0%)	4 (12.3%)	0.99
Current	9 (33.3%)	6 (30.0%)	11 (35.5%)	0.99

Abbreviations: SCLC = small cell lung cancer; NSCLC = non-small cell lung cancer.

Supplementary Table 2. The biochemical pathways, VIP scores, k-mean cluster and LASSO frequency of the selected 5 diagnostic metabolites for SCLC in males.

Metabolites	Biochemical pathways	VIP scores in the PLS-DA model	K-means clusters	Frequencies using LASSO modeling
PI(18:0/18:0)	Phospholipids metabolism	3.13	2	70%
Cer(d18:1/22:0 OH)	Sphingolipids metabolism	2.81	5	70%
Cholic acid	Bile acids metabolism	2.59	3	70%
2-arachidonylglycerol	Endocannabinoid metabolism	2.57	4	70%
IMP	Purine metabolism	2.11	2	70%

Abbreviations: IMP = inosine monophosphate; Cer = Ceramide; PI = Phosphatidylinositol; LASSO = least absolute shrinkage and selection operator; PLS-DA = partial least square discriminant analysis; SCLC = small cell lung cancer; VIP = variance in projection.

Supplementary Table 3. The biochemical pathways, VIP scores, k-mean cluster and LASSO frequency of the selected 5 diagnostic metabolites for SCLC in females.

Metabolites	Biochemical pathways	VIP scores in the PLS-DA model	K-means clusters	Frequencies using LASSO modeling
PE(18:1/20:4)	Phospholipids metabolism	2.87	5	64%
5-Methyltetrahydrofolic acid	One-carbon metabolism	2.85	3	64%
Desmosterol	Steroid metabolism	2.36	2	60%
4,5-Dihydroorotic acid	Pyrimidine metabolism	2.12	1	60%
9-HETE	Eicosanoid metabolism	2.64	4	60%

Abbreviations: 9-HETE = 9-hydroxyeicosatetraenoic acid; LASSO = least absolute shrinkage and selection operator; PE = phosphatidylethanolamine; PLS-DA = partial least square discriminant analysis; SCLC = small cell lung cancer; VIP = variance in projection.

Supplementary Table 4. The multinomial logistic regression analysis of the 5 diagnostic metabolites for SCLC in males.

Metabolites	Multinomial logistic regression coefficients	P value	Odds ratio for SCLC (95% CI)
2-AG	0.18	0.041	1.19 (1.01 - 1.37)
Cholic acid	0.91	0.006	2.48 (2.19 – 2.76)
PI (18:0/18:0)	0.51	0.01	1.61 (1.13 - 1.85)
IMP	0.23	0.034	1.26 (1.06 - 1.89)
Cer (d18:1/22:0 OH)	0.47	0.025	1.59 (1.41 - 1.78)

Abbreviations: 2-AG = 2-arachidonylglycerol; PI = Phosphatidylinositol; IMP = inosine monophosphate; Cer = Ceramide; SCLC = small cell lung cancer; CI = confidence interval.

Supplementary Table 5. The multinomial logistic regression analysis of the 5 diagnostic metabolites for SCLC in females.

Metabolites	Multinomial logistic regression coefficients	P value	Odds ratio for SCLC (95% CI)
PE (18:1/20:4)	0.85	0.017	2.34 (2.19 - 2.48)
5-Methyltetrahydrofolic acid	0.33	0.035	1.39 (1.11 - 1.67)
Desmosterol	1.21	0.002	3.35 (3.06 - 3.63)
4, 5-Dihydroorotic acid	0.95	0.005	2.59 (2.31 - 2.87)
9-HETE	0.45	0.031	1.56 (1.27 - 1.84)

Abbreviations: CI = confidence interval; SCLC = small cell lung cancer; PE = phosphatidylethanolamine; 9-HETE = 9-Hydroxyeicosatetraenoic acid.

Supplementary Table 6. The validation of the developed discriminant models using another independent cohort.

Gender	Discriminant models	Accuracy (%) (95% CI)	Sensitivity (%) (95% CI)	Specificity (%) (95% CI)	Positive predictive value (95% CI)	Negative predictive value (95% CI)
Male	PI(18:0/18:0), Cer(d18:1/22:0 OH), 2-Arachidonylglycerol, IMP and Cholic acid	91.3 (74.1-97.1)	86.6 (59.5-98.3)	84.1 (63.9-95.5)	76.5 (56.4-89.1)	91.3 (74.4-97.5)
Female	PE(18:1/20:4), 5-Methyltetrahydrofolic acid, Desmosterol, 4,5-Dihydroorotic acid and 9-HETE	81.3 (54.4-95.9)	86.4 (65.1-97.1)	84.2 (68.8-93.4)	81.3 (59.6-92.7)	86.4 (69.3-94.7)

Abbreviations: 9-HETE = 9-hydroxyeicosatetraenoic acid; Cer = Ceramide; PI = Phosphatidylinositol; CI = confidence interval; IMP = inosine monophosphate; PE = phosphatidylethanolamine.
